# Latent profile analysis of blood marker phenotypes and their relationships with clinical pain and interference reports in people with acute musculoskeletal trauma

**DOI:** 10.1080/24740527.2020.1870102

**Published:** 2021-02-16

**Authors:** Joshua Y. Lee, David M. Walton

**Affiliations:** Faculty of Health Sciences, Western University, London, Ontario, Canada

**Keywords:** chronic pain, biomarker, biopsychosocial, rehabilitation, musculoskeletal, trauma

## Abstract

**Background**: The prevalence of inadequate treatments for chronic pain has necessitated the search for biological factors that influence the transition to chronicity.

**Methods**: Antecubital blood was drawn from those who experienced acute, noncatastrophic musculoskeletal trauma. Follow-up occurred at 1, 3, 6, and 12 months with the primary outcome being Brief Pain Inventory (BPI) Functional Interference scores. Eight markers were chosen for latent profile analysis: brain-derived neurotrophic factor (BDNF); transforming growth factor-beta 1 (TGF-β1); C-reactive protein (CRP); tumor necrosis factor-alpha (TNF-α); interleukins (ILs) 1-beta, 6, and 10; and the stress hormone cortisol.

**Results**: Mean age of the 106 participants was 44.6 years and 58.5% were female. The final model indicated a three-class solution that could be adequately described by three of the eight markers: class 1 = low concentration of all markers (33.9% of the sample), class 2 = average concentration of all markers (47.7%), and class 3 = high concentration of BDNF and TGF-β1 (18.3%). BPI Pain Interference scores captured at both inception and 6-month follow-up were compared across the three groups. Mean scores were significantly higher in class 3 for the BPI Interference subscale at inception (27.0 [SD 16.4] vs. 35.8 [SD 17.3], *P* = 0.05) and at 6-month follow-up (2.2 [SD 4.8] vs. 7.3 [SD 10.7], *P* = 0.03) compared to those of the other two classes.

**Conclusions**: Although recovered populations are not significantly different in BDNF and TGF-β1 levels, those who experience persisting disability are more likely to have moderate to high levels in serum.

## RÉSUMÉ

**Contexte**: La prévalence des traitements inadéquats pour la douleur chronique a nécessité la recherche des facteurs biologiques qui influencent le passage à la chronicité.

**Méthodes**: Du sang du pli du coude a été prélevé sur des personnes ayant subi des traumatismes musculo-squelettiques aigus non invalidants. Le suivi a eu lieu à 1, 3, 6 et 12 mois avec les scores d’interférence fonctionnelle du Questionnaire concis de la douleur (QCD) comme résultat principal. Huit marqueurs ont été choisis pour l’analyse du profil latent : le facteur neurotrophique dérivé du cerveau (BDNF); le facteur de croissance transformant-bêta 1 (TGF-β1); la protéine C-réactive (CRP); le facteur de nécrose tumorale alpha (TNF-a); les interleukines (IL) 1-bêta, 6 et 10; et le cortisol, l’hormone du stress.

**Résultats**: L’âge moyen des 106 participants était de 44,6 ans et 58,5% étaient des femmes. Le modèle final a indiqué une solution à trois classes qui pourrait être correctement décrite par trois des huit marqueurs : classe 1 = faible concentration de tous les marqueurs (33,9 % de l’échantillon), classe 2 = concentration moyenne de tous les marqueurs (47,7 %), et classe 3 = concentration élevée de BDNF et TGF-β1 (18,3 %). Les scores d’interférence de la douleur BPI relevés à la fois au début et au suivi à 6 mois ont été comparés entre les trois groupes. Les scores moyens étaient significativement plus élevés dans la classe 3 pour la sous-échelle d’interférence BPI au début (27,0 [SD 16,4] comparativement à 35,8 [SD 17,3], p = 0,05) et à 6 mois de suivi (2,2 [SD 4,8] comparativement à 7,3 [SD 10,7], P = 0,03) par rapport à ceux des deux autres classes.

**Conclusions**: Bien que les populations rétablies ne soient pas significativement différentes en ce qui a trait aux niveaux de BDNF et de TGF-β1, celles qui souffrent d’une incapacité persistante sont plus susceptibles d’avoir des taux sériques modérés à élevés.

## Introduction

Chronic pain represents a substantial burden on patients and health systems, due in part to its complexity and resistance to traditional medical and pharmaceutical treatments.^1^ Though progress in interdisciplinary care strategies has been made, effective pain management remains a unique challenge.^2^ With chronic pain becoming a problem of epidemic proportions,^3^ health care researchers and providers have turned their attention toward the identification of mechanisms for early detection and intervention.^4,5^

Longitudinal modeling studies in both clinical^6^ and population-level^7^ samples have identified trajectories of pain and recovery that most commonly indicate that 15% to 25% of participants report long-term, chronic, or persistent pain and functional interference after musculoskeletal trauma seemingly regardless of the body region affected.^6,8–11^ In a previous study^12^ we identified a three-trajectory model of functional recovery from musculoskeletal trauma representing trajectories of rapid recovery (32.0% of the sample), delayed recovery (26.7%), and minimal or no recovery (41.3%). To briefly summarize, these trajectories were based on disability due to pain (or “pain interference”) over the course of 12 months where rapid recovery had the lowest symptoms at baseline with near or full recovery by 3 months, delayed recovery had high symptoms at baseline with full recovery by 12 months, and minimal/no recovery had high symptoms at baseline that persisted throughout the study. Of note is that Sterling and colleagues^11^ followed posttraumatic stress outcomes and also found a qualitatively similar three-trajectory model as the best fit to the data. The identification of consistent recovery trajectories provides new opportunities to characterize predictive mechanisms.

Advances in research and technology have led to the reemergence of a search for biomarkers that may explain the onset or persistence of pain, though these have moved from traditional approaches such as static structural imaging to more dynamic “omics” approaches (e.g., genomics, transcriptomics, proteomics, metabolomics). The results of such work have been mixed, though evidence is mounting that dysfunction in some aspect of the omics cascade may represent a valuable biomarker of acute or chronic pain. In a recent review of biomarkers of low back pain,^13^ inflammatory mediators such as high-sensitivity C-reactive protein (hsCRP), tumor necrosis factor alpha (TNF-α), and interleukin 6 (IL-6) were identified as having a potential role in the acute phase of low back pain. In the chronic phase Li et al.^14^ found that IL-10 was decreased while IL-6 was increased in people with low back pain compared to matched controls. Conversely, Klyne et al.^15^ showed that IL-6 levels do not significantly differ between those with low back pain and controls. They did, however, report a significant difference in IL-6 within the low back pain group between those reporting high levels of pain and those reporting low levels of pain. These studies suggest that there may be value in exploring blood-based proteins as markers of distress and/or pain but that simple bivariate associations may not yield consistent results.

The purpose of this study was to explore a theoretical position that eight previously identified blood-based protein/hormone biomarkers will show meaningful variance in pain-related outcomes after trauma but only when considered as clusters rather than single bivariate associations. A secondary outcome was to explore the utility of the biomarker clusters for predicting previously derived clinical recovery trajectories.

## Methods

Data from this observational cohort study were drawn from the longitudinal SYMBIOME (Systematic Merging of Biology, Mental Health and Environment) databanking study (clinicaltrials.gov ID no. NCT02711085). The study was approved by the office of Human Research Ethics at Western University and the Lawson Health Research Institute (REB 106140), and written informed consent was obtained from all participants. Eligible participants were identified by emergency or acute care clinicians from an urgent care center in London, Ontario, Canada. Clinicians identified people who were within the first 3 weeks of a general, noncatastrophic musculoskeletal injury (no hospital stays beyond 24 h or surgical correction/relocation required). These injuries included (but were not limited to) motor vehicle collisions, sports injuries, work-related injuries, sprains, strains, falls, and nondisplaced fractures that did not require surgical correction. The clinician obtained consent to allow a member of the research team to approach the potential participant. After being medically discharged, a member of the research team described the study, answered questions, and enrolled and screened potential participants prior to leaving the hospital. Eligible participants were at least 18 years old, could speak and understand conversational English, were free of cognitive impairments (e.g., no Alzheimer’s dementia, Down syndrome, etc.), were free of active malignancies in the past 5 years, and had no systemic inflammatory conditions (rheumatoid arthritis, psoriatic arthritis, scleroderma, or lupus). In addition, those who had a concussion or hospitalization in the 6 months prior to enrollment were excluded. Those under the influence of drugs or alcohol or who were otherwise not able to provide informed consent and those with no fixed address were excluded from the study.

Two samples of antecubital blood were drawn into 4 mL K2 EDTA BD vacutainer tubes by a trained phlebotomist and immediately stored on ice for transfer and storage at an immunity and proteomics lab. Prior to freezing, the samples were centrifuged for 10 min at 2000 × *g*, had plasma pipetted into up to 6 × 50 μL aliquots, and then both supernatant and pellet were stored at −80°C. Participants were concurrently provided a paper package of self-report questionnaires that included demographic metadata (age, sex, education level, work status, household income, preexisting pathology, preexisting pain, medications, body mass index, and region of injury) and pain intensity/severity and functional interference through the Brief Pain Inventory (BPI).^16^ These packages could be completed at the participant’s home within 3 to 5 days and were then exchanged for the subsequent follow-up packages upon collection of the next biological sample.

Follow-up occurred at 1, 2, 3, 6, and 12 months after injury, with the biological samples collected at baseline and 3, 6, and 12 months only. Participants were paid up to US$300 in total compensation for participation. For the purposes of this study, only the baseline blood samples were analyzed and interpreted for biomarker classes and owing to attrition, recovery up to the 6-month follow-up was used as the final end point. Functional recovery was measured using the Pain and Interference subscales of the BPI. The BPI is one of the most widely used pain interference scales globally^17^ and has adequate evidence of validity across many clinical populations, including those with musculoskeletal pain.^18^ Although the BPI has not been validated under these specific conditions of acute pain, it has been validated for postoperative pain, which represents a form of acute pain and trauma. Moreover, because this work represents a longitudinal study, the BPI afforded a certain versatility in the event of chronic pain development.

### Analysis of Serum Biomarkers

The target markers for this analysis were those shown previously to be associated with pain, distress, or inflammation.^19–25^ Through a collaborative consultative process (primarily literature review in relevant domains including pain physiology, immunology, psychology, and endocrinology, supplemented by discussions with various field experts to confirm), eight markers were specifically chosen: brain-derived neurotrophic factor (BDNF); transforming growth factor-beta 1 (TGF-β1); C-reactive protein (CRP); tumor necrosis factor-alpha (TNF-α); interleukins (ILs) 1-beta, 6, and 10; and the stress hormone cortisol. Analyte concentrations in plasma were assayed using multiplexed biomarker immunoassay kits according to manufacturers’ instruction for BDNF (Human Premixed Multi-Analyte Kit, R&D Systems Inc., cat. no. LXSAHM), TGF-β1 (TGFΒ1 Single Plex Magnetic Bead Kit, EMD Millipore, cat. no. TGFΒ1MAG-64 K-01), IL-1β, IL-6, and IL-10, and TNF-α (Human High Sensitivity T Cell Magnetic Bead Panel Multiplex Kit, EMD Millipore, cat. no. HSTCMAG-28SK). A BioPlex 200 readout System was used (Bio-Rad Laboratories, Hercules, CA), which uses Luminex xMAP fluorescent bead-based technology (Luminex Corporation, Austin, TX). Levels were automatically calculated from standard curves using Bio-Plex Manager software (v4.1.1, Bio-Rad).^26^ Cortisol (Cortisol Enzyme Immunoassay Kit, Arbor Assays, cat. no. K003-H1/H5) and CRP (C-Reactive Protein (human) ELISA Kit, Cayman Chemical Company, cat. no. 10011236) were assayed following industry standard approaches for enzyme-linked immunosorbant assay. All assays were performed in duplicate and the value for analysis was the mean concentration of the two runs.

## Analysis

Participant characteristics were summarized descriptively (means and distributions or proportions).

### Pre-analysis of Analytes

Prior to primary analyses we explored the distribution of the data both qualitatively and statistically. Concentrations of all eight analytes were significantly positively skewed and in violation of normality via Kolmogorov-Smirnov tests. All concentrations were then square root transformed to reduce skewness and create normally distributed data. High outliers (>4 SD above the mean; identified after normalization of the data) or those for which the assay resulted in nondetectable (too low or too high) concentrations were then removed. Beyond 4 SD represents 0.1% of the population, which may be important but is not likely to be clinically feasible to address. Data were then *z*-transformed to place all concentrations on the same scale with a mean of 0.0 and standard deviation of 1.0.

### Bivariate Associations

A matrix of all cross-product Pearson correlations between the eight markers was created as an exploratory step and to identify potential problems with collinearity in cluster analysis (*r* > 0.80). There was no statistical correction for multiple comparisons, accepting the potential for alpha error rather than prematurely rejecting potentially important findings at this exploratory stage. If any significant correlations were identified, biomarkers were regressed against each other in an iterative fashion in order to determine the variance inflation factor as a result of the correlations.

### Profile Analysis

Meaningful clusters in the data were identified with maximum likelihood estimation (MLE)-based latent profile analysis (LPA) as previously described^27^ using MPlus software v6.12 (Muthen and Muthen, Los Angeles, CA).^28^ In brief, MLE-based analysis involves creating a model that accurately represents the data. However, unlike general analysis of variance (ANOVA)-based methods that rely on sample means to develop a linear model, MLE-based analysis relies instead on the probabilities generated by each of the individual data points. Each of these individual probabilities are then used to calculate a distribution that most likely fits the available data. Because of this data-driven approach to probability generation, this method is also robust against missing data points.^29^ Using all eight target biomarkers, a series of models was constructed, starting with a single profile (termed “class”) and increasing until model fit no longer improved in a meaningful way, the LPA estimation could no longer derive a mathematically definable model, one of the latent classes possessed fewer than 10% of participants, or the class structure did not make clinical sense. The fit indicators of interest were the Akaike information criterion (AIC),^30–32^ the Bayesian information criterion (BIC),^30–32^ entropy,^31^ and the adjusted Lo-Mendell-Rubin likelihood ratio test (LMR-LRT)^30,32^ while considering solutions that provide generally strong posterior classification probabilities (ideally ≥0.85). Though no set criteria exist for deeming model fit acceptable,^32^ the cluster solution that provides the lowest AIC and BIC and the highest entropy value (acceptable >0.70, ideal >0.80) that also conforms to theory is generally considered optimal.^33^ The LMR-LRT is used to statistically compare the fit of the *k* cluster solution with that of the *k* − 1 class solution. When fit no longer statistically improves (*P* > 0.05) with the addition of a new class, the solution with the smaller number of classes is generally accepted.^32,34^

In the interest of parsimony, once an overall class solution was determined, biomarkers were systematically eliminated to obtain the simplest discriminatory model. To start, mean differences in square root–transformed marker concentration were explored across the identified classes using one-way ANOVA. The marker with the smallest interclass differences was eliminated first, followed by the next smallest, and so on until the simplest model remained that still showed good fit indicators in LPA. The intention was that each of the blood markers defining the final class solution should show a significant difference between the groups.

### Recovery and Outcome Analysis

After LPA, each participant was assigned to one of the identified classes based on relative blood marker concentration. From a previous study^12^ of derivation of recovery curves, each participant was also assigned to one of three trajectory classes: rapid, delayed, or minimal recovery. Both the rapid and delayed recovery groups were grouped together as a “recovery predicted” group and proportions of the blood marker clusters were statistically compared against the “minimal or no recovery predicted” group using chi-square analysis.

### Sample Size Estimation

There is little guidance in the literature for optimal sample size in MLE-based LPA. Prior to the exploratory analyses described herein there was also no clear existing evidence to inform the likely number of clusters or the relative proportions or communalities to assist with sample estimation. Therefore, we adopted the general position in the field that a minimum of 100 samples is a minimum for meaningful results and continued to position the analyses as exploratory in nature; that is, hypothesis generating rather than hypothesis testing.

## Results

Table 1 provides the characteristics of the study population. During the 36 months of the study, a trained research associate spent the first half of that time recruiting from the urgent care center during regular daytime hours. During this time, a total of 345 eligible participants were identified, of whom 183 (53% recruitment) consented to participate. Of these 183 participants, only 120 (78%) provided enough data for baseline analysis and only 5 (4%) were missing primary outcome data. There were 109 participants in the SYMBIOME database who provided blood samples within 3 weeks of musculoskeletal trauma. After assay, data for 3 participants were removed because all analytes were not detectable or out of range of the kits. Mean age of the remaining 106 participants was 44.6 years and 58.5% of the sample was female. The modal mechanism of injury was reported as “other” and 74.3% of the sample reported the primary region of injury as the upper or lower extremity (vs. the axial spine). Pain severity and interference at inception were moderate (mean severity = 4.5/10, SD 2.0; mean interference = 28.6/70, SD 16.8). A combination of Kolmogorov-Smirnov test, skewness and kurtosis values, and a visual inspection of histograms, normal Q-Q plots, and box plots showed that the biomarker concentrations were approximately normally distributed for each marker. Kolmogorov-Smirnov values were significant (*P* < 0.05) for TNF-α, cortisol, and CRP, but the absolute *z*-values of their corresponding skewness and kurtosis statistics were within an acceptable range for our sample size (−3.29 < *z* < 3.29).^35^ This along with the abovementioned indicators suggested that the data did not display a significant departure from normality. Results of the iterative regression of biomarkers also revealed that multicollinearity was not likely to be a problem because each of the variance inflation factor values was below a conservative cut score of 3,^36^ with no value exceeding 2.6.

**Table 1.** Characteristics and baseline values of SYMBIOME participants in this analysis (*N* = 109)

Sex (% female)	58.5%
Age (mean, range)	44.6 years (18–66)
Body mass index (mean, range)	26.4 kg/m^2^ (14.4–51.5)
Primary region of injury (%) Axial Extremity	25.7% 74.3%
Mechanism of injury (%) Motor vehicle injury Fall Hit by person or object Awkward lift or twist Other	7.1% 28.6% 19.4% 14.3% 30.6%
Brief Pain Inventory at inception (mean, range) Pain severity (/10) Pain interference (/70)	4.5 (0–8) 28.6 (0–67)

Table 2 provides the cross-product correlation matrix between all biomarker pairs after removal of outliers and square root transformation. BDNF and TGF-β1 demonstrated the strongest association (*r* = 0.74, *P* < 0.01). Cortisol and CRP did not appear to be associated with any other biomarker, whereas IL-6 and IL-1β were significantly correlated with all markers except those two.

**Table 2.** Cross-product correlation matrix of all eight analytes (Pearson’s *r*) after square root transformation

	IL-6	IL-10	TNF-α	TGF-β1	BDNF	CRP	Cortisol
IL-1β	0.47**	0.53**	0.42**	0.34**	0.31**	0.03	−0.06
IL-6		0.47**	0.34**	0.25*	0.21*	−0.01	0.01
IL-10			0.42**	0.19*	0.17	−0.09	−0.10
TNF-α				−0.01	0.18	0.02	0.11
TGF-β1					0.74**	−0.16	0.11
BDNF						−0.01	0.16
CRP							−0.05

*Correlation is significant at *P* < 0.05.

**Correlation is significant at *P* < 0.01.

IL-6 = interleukin 6; IL-10 = interleukin 10; TNF-α = tumor necrosis factor alpha; TGF-β1 = transforming growth factor-beta 1; BDNF = brain-derived neurotrophic factor; CRP = C-reactive protein; IL-1β = interleukin 1-beta.

**Figure 1.**
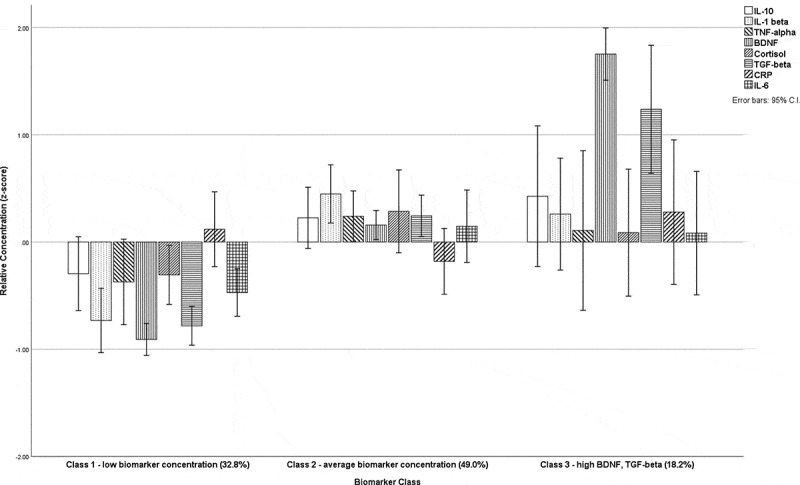
Graphical representation of the three-class latent profile solution along with the frequencies of each class. All eight target markers presented in a three-class profile solution were labeled accordingly: class 1 = low biomarker concentration (32.8% of the sample), class 2 = average biomarker concentration (49.0%), class 3 = high BDNF and TGF-β1 (18.2%). Relative concentration represents *z*-transformed values

**Figure 2.**
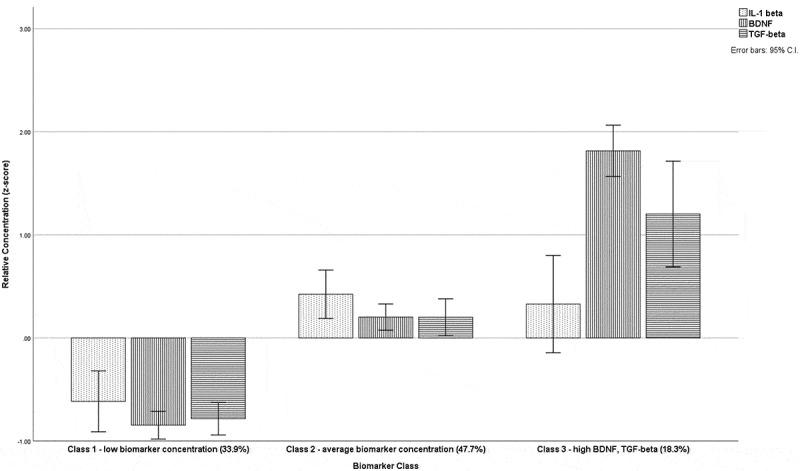
Graphical representation of the three-class latent profile solution adequately described by three of the eight markers. Classes were labeled accordingly: class 1 = low biomarker concentration (33.9% of the sample), class 2 = average biomarker concentration (47.7%), and class 3 = high BDNF and TGF-β1 (18.3%). Relative concentration represents *z*-transformed values

Table 3 shows the results of the LPA models with associated fit indicators for the models tested. The final class solution was a three-class model because it showed a meaningful improvement over a two-class solution based on relevant fit indicators (AIC = 2257.31, BIC = 2348.82, entropy = 0.83, LMR-LRT = 28.08, *P* = 0.08). Figure 1 show the relative concentrations of all eight markers in the three-class model. Although a four-class model did provide an improved fit according to the listed indicators, one of the identified classes contained less than 10% of the sample and was therefore excluded in accordance with a priori decisions on class identification. After settling on the three-class model, analytes were removed in a systematic fashion based on total interclass differences. CRP, *F*(2,108) = 0.14, *P* = 0.87, and cortisol, *F*(2,108) = 2.34, *P* = 0.10, displayed the smallest interclass mean differences (Figure 1) and were eliminated first. Table 3 also shows the model fit adjustment of the three-class latent profile solution with the sequential elimination of biomarkers. TNF-α, *F*(2,108) = 10.65, *P* < 0.01, IL-6, *F*(2,108) = 6.06, *P* < 0.01, and IL-10 were also removed, in that order, each time retesting model fit and posterior classification probabilities. The remaining three markers were BDNF, TGF-β1, and IL-1β. BDNF and TGF-β1 were both discriminative across the three classes, and IL-1β provided improved discrimination between the two lower concentration classes. The decision to retain IL-1β despite acceptable model fit is described in the Discussion section. The final model indicated a three-class solution that could be adequately described by three of the eight markers (AIC = 827.41, BIC = 865.09, entropy = 0.80, LMR-LRT = 34.08, *P* = 0.03). The three classes were labeled according to the relative concentrations of the three markers as follows: class 1 = low concentration of all markers (33.9% of the sample), class 2 = average concentration of all markers (47.7%), and class 3 = high concentration of BDNF and TGF-β1 (18.3%). The three-class model provided strong probabilities of class assignment according to the LPA model calculation, where probability of correct identification for class 1 was 90.8%, for class 2 it was 90.3%, and for class 3 it was 88.5%. Figure 2 shows relative (*z*-transformed) concentrations graphically and Table 4 shows the raw (nontransformed) values with 95% confidence intervals.

**Table 3.** Fit indicators for latent profile analysis and class assignment: AIC, BIC, entropy, and LMR-LRT

Model	AIC	BIC	Entropy	LMR-LRT (*P*)
Two-class	2298.77	2366.06	0.78	90.80 (0.07)
Three-class	**2257.31**	**2348.82**	**0.83**	**58.08 (0.08)**
Four-class	2231.06	2346.79	0.89	43.23 (0.30)
Three-class (CRP removed)	1986.71	2067.45	0.83	57.81 (0.058)
Three-class (cortisol removed)	1678.22	1748.20	0.82	56.22 (0.054)
Three-class (TNF-α removed)	1384.94	1444.15	0.81	47.06 (0.053)
Three-class (IL-6 removed)	1121.54	1169.99	0.80	39.44 (0.029)
Three-class (IL-10 removed)	**827.41**	**865.09**	**0.80**	**34.08 (0.033)**
Three-class (IL-1β removed)	539.24	566.16	0.81	27.44 (0.025)

Values highlighted in bold indicate the preferred class for analysis.

AIC = Akaike information criterion; BIC = Bayesian information criterion; LMR-LRT = Lo-Mendell-Rubin likelihood ratio test; CRP = C-reactive protein; TNF-α = tumor necrosis factor alpha; IL-6 = interleukin 6; IL-10 = interleukin 10; IL-1β = interleukin 1-beta.

**Table 4.** Mean (raw, untransformed) concentrations of the three retained analytes across the three classes identified through LPA

	Overall mean (95% confidence interval)	Class 1 (*n* = 42)	Class 2 (*n* = 47)	Class 3 (*n* = 20)	*F* (*P*)
**IL-1β (pg/mL)**	2.71 (2.43, 2.99)	1.32 (1.07, 1.58)	3.46 (3.14, 3.77)	3.19 (2.52, 3.87)	19.75 (<0.01)^a^
**BDNF (ng/mL)**	3.55 (3.00, 4.09)	1.78 (1.22, 2.34)	3.08 (2.71, 3.46)	8.65 (7.51, 9.80)	182.92 (<0.01)^b^
**TGF-β1 (ng/mL)**	24.45 (21.11, 27.79)	16.96 (12.22, 21.70)	21.78 (19.02, 24.54)	46.67 (35.74, 57.60)	67.14 (<0.01)^b^
IL-10 (pg/mL)	21.12 (18.08, 24.16)	15.7 (11.9, 19.5)	23.1 (18.5, 27.7)	27.8 (18.2, 37.4)	6.06 (<0.01)^a^
IL-6 (pg/mL)	92.17 (80.05, 104.29)	70.1 (56.9, 83.2)	101.9 (81.5, 122.2)	115.6 (80.3, 150.8)	4.81 (0.01)^a^
TNF-α (pg/mL)	5.61 (5.08, 6.13)	4.9 (3.9, 5.8)	6.0 (5.4, 6.7)	6.1 (4.6, 7.5)	2.77 (0.07)
CRP (mg/L)	3.34 (2.65, 4.01)	3.22 (2.24, 4.21)	3.36 (2.29, 4.44)	3.48 (1.41, 5.54)	0.00 (1.00)
Cortisol (μg/dL)	12.04 (10.58, 13.49)	10.44 (8.65, 12.22)	13.42 (10.75, 16.08)	12.05 (8.68, 15.43)	1.99 (0.14)

Statistical tests were one-way analysis of variance with Tukey’s post hoc test using square root–transformed data to reduce deviations from normality. The three markers retained in the final model solution are shown in bold.

^a^The mean concentration was significantly lower in class 1 compared to the other two groups.

^b^The mean concentrations of both BDNF and TGF-β1 were significantly different across all three groups.

LPA = latent profile analysis; IL-1β = interleukin 1-beta; BDNF = brain-derived neurotrophic factor; TGF-β1 = transforming growth factor-beta 1; IL-10 = interleukin 10;

IL-6 = interleukin 6; TNF-α = tumor necrosis factor alpha; CRP = C-reactive protein.

With each participant assigned to the most likely biomarker class based on posterior probabilities, the sample was split into three groups. BPI Pain Severity and Pain Interference scores captured both at inception (<3 weeks after injury) and at 6-month follow-up were compared across the three groups using one-way ANOVA. Significant main effects were present in each of the 6-month follow-up scores, and a strong trend (*P* = 0.06) was seen in the main effect of class for the BPI Pain Interference score at inception (Table 5). The pattern of responses indicated that the scores for class 3 (high BDNF/TGFβ1) were higher than those of the other two classes. As such, post hoc tests were conducted with the scores of the first two classes grouped (low or average concentration of all markers) against those of class 3, using a Mann-Whitney *U* test because of skewed data at the 6-month follow-up. Mean scores were significantly higher in class 3 for the BPI Interference subscale at inception (27.0 [SD 16.4] vs. 35.8 [SD 17.3], *P* = 0.05) and at 6-month follow-up (2.2 [SD 4.8] vs. 7.3 [SD 10.7], *P* = 0.03) compared to those of the other two classes, and BPI Pain severity at 6 months showed a strong trend toward significance (0.3 [SD 0.7] vs. 1.4 [SD 1.8], *P* = 0.07).

**Table 5.** Mean scores on the BPI Pain Severity and Pain Interference scales, captured at inception (<3 weeks after injury) and at 6-month follow-up, separated by biomarker class

	Class 1 (low all markers)	Class 2 (average all markers)	Class 3 (high BDNF/TGF-β1)	*F* (*P*)
BPI Pain Severity (acute)	4.9 (4.2, 5.5)	4.1 (3.6, 4.7)	4.6 (3.6, 5.6)	1.52 (0.23)
BPI Pain Interference (acute)	30.6 (24.7, 36.4)	23.9 (19.5, 28.3)	33.7 (25.0, 42.3)	2.9 (0.06)
BPI Pain Severity (6 months)	0.2 (0.1, 0.4)	0.4 (0.0, 0.8)	**1.3 (0.0, 2.7)**	**3.9 (0.03)**
BPI Pain Interference (6 months)	1.8 (0.9, 2.8)	2.7 (0.9, 4.4)	**6.1 (1.4, 10.8)**	**3.4 (0.04)**

BPI = Brief Pain Inventory; BDNF = brain-derived neurotrophic factor; TGF-β1 = transforming growth factor-beta 1. Bold values represent significant main effects at 6-month follow-up.

Although we did capture pre-existing pain as a separate construct, many participants did not report their specific diagnosed conditions, but they did report their medications. For our current study, the pre-existing pain conditions were determined either by self-report or by extrapolating from their primary prescriptions. Becuase this represented a potentially influential factor to the results, we performed a chi-square analysis of preexisting pain against our three-class biomarker model. This yielded a nonsignificant chi square value because there did not seem to be any significant difference between those who had a preexisting pain condition and those who did not (χ^2^ = 5.07, *P* = 0.08).

## Discussion

We have presented a first step toward derivation of a potentially useful panel of immunological, neurotrophic, and endocrine markers assayed from serum for use in posttraumatic pain research. Through a multistep approach to latent profile analysis, a three-class solution was identified that could be adequately described by three of eight markers (BDNF, TGF-β1, and IL-1β), though at least two other markers (IL-6 and IL-10) also showed some significant discriminative accuracy between the classes. Further, participants assigned to the class representing the highest mean BDNF and TGF-β1 concentrations also tended to rate higher on self-rated scales of pain-related functional interference when measured <3 weeks after noncatastrophic musculoskeletal trauma or 6 months posttrauma.

As shown in Figures 1 and 2, an argument could have been made for removing IL-1β from the final model and retaining only TGF-β1 and BDNF, though the strong correlation between these two markers (Table 2) led us to retain a third marker for better discriminative accuracy between class 1 and class 2 and to allow greater opportunities for exploration of potential mechanisms behind the biomarker/clinical outcome associations found here. Both IL-10 and IL-6, and to a lesser extent TNF-α, could also have been retained because they also discriminated between the two lower concentration classes, but IL-1β provided the greatest discriminative accuracy (largest between-class mean difference) and was therefore chosen as the third marker. To our knowledge this is the first time that these three markers (arguably, up to six markers) have been shown to interact as a panel that may have clinical utility if the findings can be replicated in an independent sample. It is notable that the only two markers that showed no between-class differences (CRP and cortisol) were also those that showed no meaningful association with any of the other six markers (Table 2). This should not be mistaken as indicating that these markers are unimportant in research into pain and trauma but rather that through cluster analysis they did not contribute important explanatory utility to the classes identified herein.

BDNF is a small peptide that is involved in myriad of functions related to survival, growth, and plasticity of neurons and it acts as a key regulator of learning and memory.^37^ It carries out this activity by binding to its receptor tyrosine kinase B (TrkB) and activating signaling cascades involved in gene transcription for proteins of stress and plasticity.^37–39^ TGF-β1 is a ubiquitous pleiotropic cytokine that, along with its immunomodulatory function, is involved in cell growth, development, angiogenesis, and wound healing.^40^ TGF-β1 has been shown to play a role in the long-term facilitation of neuronal activity and transmission.^41^ Both BDNF and TGF-β1 do not seem to display any significant short-term effects on sensory neurons, but they appear to have a role in facilitating long-term signaling by affecting new growth at sensory neuron synapses.^41,42^ With regard to pain, Sikandar and colleagues have demonstrated that primary afferent-derived BDNF may be involved in the transition from acute to chronic pain. By applying an inflammatory stimulus to mice, they showed that conditional BDNF knockout mice do not develop an ongoing mechanical hyperalgesia.^25^ Similarly, Richner and colleagues have shown that BDNF, via TrkB receptors, can reduce inhibition at the spinal dorsal horn by downregulating the expression of a protein known as KCC2.^43^ By inhibiting this BDNF-regulated pathway, they were able to prevent the decrease in KCC2 and impair mechanical allodynia. TGF-β1, with its ability to suppress immune activity and promote endogenous opioid signaling, appears to have a protective effect against the development of chronic neuropathic pain.^44^ The association between BDNF and TGF-β1 appears to have prior empirical support, at least in animal models. Sometani et al. have shown that TGF-β1 administered to cortical neurons of the rat increases BDNF and TrkB expression, suggesting that BDNF may require TGF-β1 in order to carry out its neurotrophic effects.^45^ Both BDNF and TGF-β1 also appear to regulate the *Gadd45* family of enzymes, which have been implicated in psychiatric diseases.^46^ Although it is unclear in what capacity BDNF and TGF-β1 exert their influence in persistent disability and pain in humans, their association is at least biologically plausible.

Although IL-1β does not offer much in the way of computational discrimination between average and high concentrations (i.e., between class 2 and class 3), it still represents a useful element in the model. Not only does it provide greater accuracy in distinguishing from the low biomarker concentrations (class 1) but it also provides some potential insight into the effects of trauma. It was found that an elevation in glucocorticoid levels contributed to conditioned fear memory in rats and that this was potentially an IL-1β-mediated event. In the aforementioned study, blocking or enhancing IL-1β signaling resulted in decreased or increased fear memory, respectively.^47^ In a separate study, mice exposed to controlled bouts of severe stress demonstrated an enhanced fear learning, which was attenuated with the inhibition of IL-1β signaling.^48^ Human studies around this topic have yet to provide consistent results, but there appears to be a potential role for IL-1β in the development of posttraumatic stress disorders.^49^ Together, these studies suggest that IL-1β may be a useful target when considering overall risk for the development of persistent symptoms. IL-1β and IL-10 (i.e., the next biomarker candidate) appear to behave in a similar manner throughout the three classes, and this may be due to the finely tuned mechanisms of inflammation. An aggressive physiological response, inflammation is closely regulated in order to prevent the development of chronic complications. The mechanism of action tends to be through a dynamic process that occurs alongside resolution, rather than in a strict on–off fashion.^50^ It has been shown that a controlled program of resolution is activated within hours of an inflammatory response, possibly in a tissue-specific manner, and that these processes can occur simultaneously on different gradients in order to regulate repair and restoration.^50,51^ Given the timing of these events, it is not unreasonable to see the levels of both pro- and anti-inflammatory factors coinciding with one another, especially because these biomarkers were taken from people within the first 3 weeks of their trauma. However, for reasons of parsimony, IL-1β appears to be a more promising candidate for risk prognostication at this time compared to IL-10.

Despite the significance of BDNF and TGF-β1, at this early stage of research it is advised that future studies consider incorporating all of the biomarkers explored here. Cytokines often act synergistically such that their effectiveness is substantially increased when working in concert with one another.^52^ Together they can affect multiple systems through peripheral and central cross-talk mechanisms to influence immune, endocrine, and neuronal functioning.^53,54^ For example, previous work by Sterling and colleagues demonstrated a potential role for both TNF-α and CRP, wherein the latter appeared to show some discriminative accuracy in identifying those with more severe symptoms following whiplash injury.^55^ Additionally, Li et al.^14^ and Klyne et al.^15^ found that IL-6 may also be involved in discriminating between control and low back pain groups and within low back pain groups, respectively.

The effects in our study may be related to the simultaneous consideration of multiple markers in the same class. Many prior studies, including a recent companion manuscript from the same data set (under review), we showed that in isolation none of the eight markers explored here were associated with clinical pain or interference levels, though several potential moderating effects of psychosocial variables were identified. We believe, however, that it is the multivariate cluster nature of the results from this latent profile analysis that will prove more valuable. In the same way that a single genetic polymorphism is unlikely to explain important variance in a clinical outcome but Gene × Gene interactions are more likely, the expression of certain proteins, at certain levels, in the same person appears as though it may be a more fruitful direction for exploration. In exploring this hypothesis, we are working at the “proteomics” level of the “omics” cascade, downstream from genomic and transcriptomic processes but upstream from metabolomics. Future research directions could use these results and then move along that cascade in either direction to further explain these findings. It is important to reiterate that this is exploratory research and needs replication and that despite some biological plausibility, association is not causation.

## Limitations

There are some important limitations of this study to consider. First, blood was drawn using venipuncture, which may involve increased anxiety for some. All participants were notified at screening and prior to consent of the requirement for repeated blood draws, which may have been sufficient to eliminate those with needle-based anxieties. Second, blood was drawn as participants presented to the urgent care center regardless of the time of day. This allowed for a more accurate “baseline” sample to be taken as close to the time of trauma as possible, but it does not take into account the known diurnal variations in some of these biomarkers, specifically cortisol^56^ and CRP.^57^ If sample collection had occurred at the same time each day, a greater overall effect of the eight-biomarker model may have been observed. Although the concentrations of the chosen biomarkers were relative to the rest of the cohort, the average concentrations were within the range of values that have been observed in other healthy adults of various ages.^58–62^ It is worth noting, however, that the average concentrations of IL-6 (92.17 pg/mL) and, to a lesser degree, IL-10 (21.12 pg/mL) were significantly higher than reported normative values. Despite this discrepancy, the concentration of IL-6 is still not beyond what is considered a normal range, because a healthy individual can experience increases up to 140 pg/mL from strenuous exercise.^63^ IL-6 present in muscle tissue has also been shown to be very sensitive to stress and injury, which can lead to significant elevations in the tissue and subsequent elevations in IL-10.^63,64^ This may be particularly relevant because this cohort was selected based on exposure to musculoskeletal trauma. Because samples were collected within 3 weeks of trauma, there is a possibility of varying degrees of inflammatory activity depending on when the sample was taken. Recruitment took place at an urgent care center during normal work hours and not at an emergency department, which may also have contributed to the consistency and relative intensity of biomarker activity. Another factor to consider is the way the samples were processed and stored. Each blood sample was stored at 4°C over a period of 1 to 2 days before being aliquoted and frozen before analysis. It has been shown that some cytokines are very sensitive to refrigeration and freeze–thaw cycles, whereas others are relatively stable.^65^ All of the blood samples were subjected to the same conditions, but this may have different effects depending on the cytokine in question. This represents another potential limitation of the study because we were unable to separate the serum and analyze the sample on the same day, which is considered to be the ideal situation.^65^ These factors may also provide an explanation as to why most of the raw concentrations of biomarkers were positively skewed. Lastly, because this was an exploratory study, we have not attempted to build more complex multivariate models, including, for example, sex, age, or psychological distress. Our previous work supports the notion that the associations shown here may be moderated by other important person-level variables that require larger data sets to properly explore. This represents an important step for future studies, because analyzing biomarker concentrations in isolation may be an oversimplification of their role in persistent pain. We also recognize that race and ethnicity have been shown to be important clinical dimensions in pain^66^; however, many of the participants chose not to report their race or ethnicity (only 19% of respondents did so). This made it impossible to stratify the data or even comment on the possible ramifications within this study, but it represents an important area of exploration for future research.

In conclusion, we have presented an exploratory study of immune, neurotrophic, and endocrine biomarkers in a population of people in the acute stage of noncatastrophic musculoskeletal trauma using latent profile analysis. Our results show that a three-class profile solution appears to be the most statistically sound. Interestingly, six out the eight biomarkers showed some potential to discriminate between different classes, with cortisol and CRP being the only exceptions. Classes were organized based on increasing serum biomarker concentration, where the third class was characterized by high BDNF/TGF-β1. Although recovered populations are not significantly different in their levels of BDNF and TGF-β1, those who experience persisting disability or pain are more likely to have moderate to high levels in serum. These findings, if used in combination with other self-report measures of pain and distress, may provide a simple biopsychosocial approach to phenotyping pain in a clinical population.
